# A comparative analysis of deep learning-based location-adaptive threshold method software against other commercially available software

**DOI:** 10.1007/s10554-024-03099-7

**Published:** 2024-04-18

**Authors:** Daebeom Park, Eun-Ah Park, Baren Jeong, Whal Lee

**Affiliations:** 1 Department of Radiology, Seoul National University Hospital, Seoul, Korea; 2 Department of Radiology, Seoul National University College of Medicine, Seoul, Korea; 3 Institute of Radiation Medicine, Seoul National University Medical Research Center, Seoul, Korea; 4 Department of Clinical Medical Sciences, Seoul National University College of Medicine, Seoul, Korea

**Keywords:** Coronary computed tomography angiography, Coronary artery disease, Automatic segmentation, Software platform, Deep learning-based location-adaptive threshold method

## Abstract

Automatic segmentation of the coronary artery using coronary computed tomography angiography (CCTA) images can facilitate several analyses related to coronary artery disease (CAD). Accurate segmentation of the lumen or plaque region is one of the most important factors. This study aimed to analyze the performance of the coronary artery segmentation of a software platform with a deep learning-based location-adaptive threshold method (DL-LATM) against commercially available software platforms using CCTA. The dataset from intravascular ultrasound (IVUS) of 26 vessel segments from 19 patients was used as the gold standard to evaluate the performance of each software platform. Statistical analyses (Pearson correlation coefficient [PCC], intraclass correlation coefficient [ICC], and Bland-Altman plot) were conducted for the lumen or plaque parameters by comparing the dataset of each software platform with IVUS. The software platform with DL-LATM showed the bias closest to zero for detecting lumen volume (mean difference = -9.1 mm^3^, 95% confidence interval [CI] = -18.6 to 0.4 mm^3^) or area (mean difference = -0.72 mm^2^, 95% CI = -0.80 to -0.64 mm^2^) with the highest PCC and ICC. Moreover, lumen or plaque area in the stenotic region was analyzed. The software platform with DL-LATM showed the bias closest to zero for detecting lumen (mean difference = -0.07 mm^2^, 95% CI = -0.16 to 0.02 mm^2^) or plaque area (mean difference = 1.70 mm^2^, 95% CI = 1.37 to 2.03 mm^2^) in the stenotic region with significantly higher correlation coefficient than other commercially available software platforms (*p* < 0.001). The result shows that the software platform with DL-LATM has the potential to serve as an aiding system for CAD evaluation.

**Supplementary Information:**

The online version contains supplementary material available at 10.1007/s10554-024-03099-7.

## Introduction

Coronary artery disease (CAD) is the leading cause of mortality worldwide and can cause various diseases related to the cardiovascular system [1–4]. Coronary computed tomography angiography (CCTA) is widely used for CAD diagnosis [5–8]. Automatic segmentation of the coronary arteries can be performed using CCTA images, and several analyses related to CAD are possible [9, 10]. Several methods are available for coronary segmentation; however, they require manual segmentation processes to generate an accurate 3D model of coronary arteries, which induces intra- and inter- observer variability [11–13]. To overcome laborious manual segmentation processes, several CCTA studies recently utilized deep learning algorithms, particularly concerning coronary artery segmentation [14–16].

The stenosis severity of the lumen is an important factor for diagnosing CAD because blood flow decreases in the stenotic region of the vessels [17, 18]. In addition, the components of plaques, including lipids and calcium in blood vessels, are important factors in the diagnosis of CAD [19, 20]. Therefore, accurate detection of the lumen or plaque volume in the stenotic region using an automatic segmentation technique is preliminarily required to provide several useful analytic tools for diagnosing CAD.

A previous study showed that the location-adaptive threshold method (LATM) could overcome the segmentation of coronary arteries in the region of stenosis [21]. Subsequently, a deep learning-based LATM (DL-LATM) was developed by combining deep learning with LATM. The performance of the software with DL-LATM for lumen or plaque segmentation was evaluated against commercially available software platforms. To compare the performance of software platforms with each other, the dataset from intravascular ultrasound (IVUS) was used as the gold standard [22, 23]. This study aimed to observe the potential of DL-LATM for serving as an aiding system for diagnosis of CAD by evaluating the overall segmentation performance and especially in the stenotic region.

## Materials and methods

The Institutional Review Board reviewed and approved the retrospective nature of this study and waived the requirement for informed consent (IRB No. 2107-192-1237). The methods for (1) subjects, (2) CCTA image acquisition and reconstruction, and (3) IVUS imaging protocol and analysis were adopted from previous research [21].

### Subjects

Thirty target coronary segments of 22 patients were included in this study. Patients with suspected or known CAD underwent CCTA and invasive coronary angiography with IVUS at Seoul National University Hospital from March 1, 2009 to June 30, 2010. The exclusion criteria were poor CCTA image quality in the target segment due to motion artifacts (*n* = 4) and segmentation failure of software platforms. The exclusion criteria for segmentation failure include the file import failure and significant measurement difference against the reference data. In the event of file import failure, coronary artery segmentation becomes impossible. If there is a significant measurement difference against the reference data, it could introduce bias in evaluating the performance of coronary artery segmentation. The detailed information including exclusion criteria and failure rates of coronary artery segmentation for different software platforms is show in Supplementary Table S1. Overall, 26 target coronary segments from 19 patients were included in this study (mean age, 64.6 years; female, 15.3%). The characteristics of target coronary segments such as the location, length, plaque volume measured in IVUS, coronary artery calcium score on CCTA, stenosis degree, and plaque burden are shown in Supplementary Table S2.

### CCTA image acquisition and reconstruction

The CCTA images were acquired following the guidelines of the Society of Cardiovascular Computed Tomography [24]. CCTA was performed using a dual- source, 16-slice, or 256-slice CT scanner (SOMATOM Definition Siemens Healthineers; Sensation 16, Siemens Healthineers; iCT, Philips Healthcare) (*n* = 17, 8, and 1, respectively). Once antecubital intravenous access was established, a dose of 70–90 milliliters of iopromide (Ultravist 370; Shering, Berlin, Germany) was given, followed by the administration of a 50 milliliters mixture containing 8 parts normal saline and 2 parts contrast medium at a flow rate of 4 milliliters per second, facilitated by a dual power injector (Stellant; Medrad, Indianola, PA, USA). CT acquisition commenced following the determination of the bolus-triggering method, which monitored the signal intensity of the contrast medium within the mid ascending aorta. The scanning began 8 s after surpassing a threshold trigger set 150 HU higher than the baseline.

The scan parameters were set as follows: (a) collimation = 32 × 0.6 mm / 16 × 0.75 mm / 128 × 0.625 mm; (b) tube voltage = 100 kVp or 120 kVp; (c) tube currents = 104–620 mA; (d) rotation times = 270–370 ms. The tube voltage and currents were set depending on the body habitus. A mono-segment reconstruction algorithm was used to generate images using a retrospective electrocardiographic-gated technique. Reconstruction parameters were set as follows: (a) slice thickness = 0.8–1 mm; (b) increments = 0.5–0.7 mm; (c) kernel = a medium soft convolution kernel. The CCTA data were collected in a motion-free manner, typically in the mid-diastolic phase.

### IVUS imaging protocol and analysis

To acquire the images using IVUS, a 40 MHz, 2.9 F catheter (Boston Scientific Scimed) was used with an axial and lateral resolution of ± 80 and ± 200 μm, respectively. IVUS was performed throughout the length of the target segment after intracoronary nitroglycerine administration. A pullback system with a standard automated motor was used for acquiring IVUS images by measuring cross-sectional area at a speed of 0.5 mm/s with 30 frames/s. A cardiologist with 15 years of experience used computerized software (EchoPlaque, INDEC Medical Systems, Inc.) to analyze the target segments of the IVUS images. To measure the lumen area, manual traces of the lumen-intima interface and external elastic membrane were performed for each cross-sectional plane. Simpson’s rule was employed to calculate the volume parameters of each lesion using cross-sectional areas. Side branches were used as initial landmarks to record the locations of the target segments for CT-IVUS matching. On the 3D volume-rendered CCTA images, the location information was provided as a reference for the target segment, including documentation of the up-or downstream direction with lesion lengths (Supplementary Table S2).

### Measurement of lumen or plaque parameters for three software platforms

**Fig. 1:**
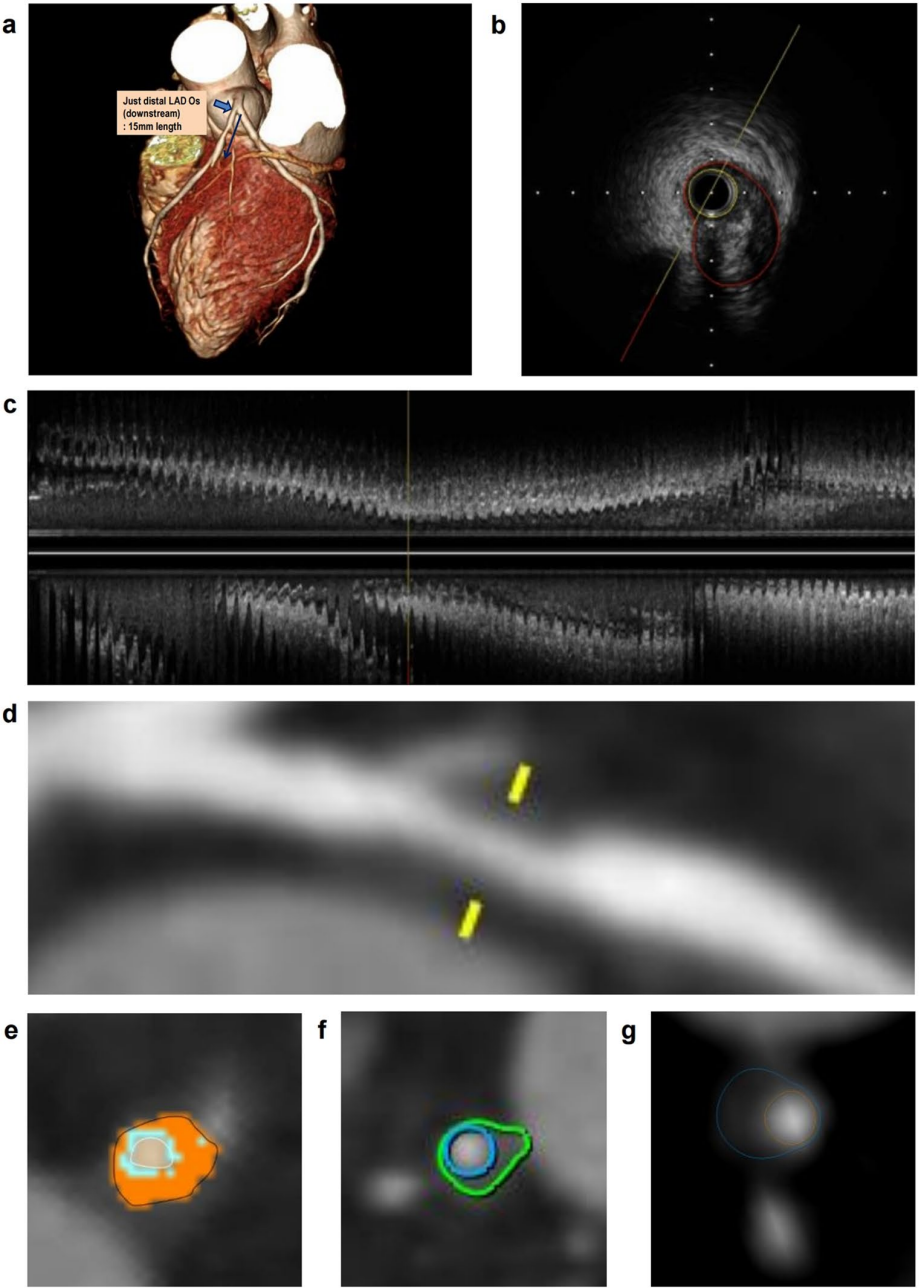
Representative images of the curved planar reformation and cross-sectional planes of the lumen or plaque region for each software platform. (**a**) The location information annotated on a three-dimensional image of the coronary computed tomography angiography that provided the reference to the exact length of the target segment. (**b**) The representative cross-sectional plane of the IVUS. (**c**-**d**) Longitudinal and curved planar reformation images of the target segment of the IVUS (**c**) and the software 1 (**d**), respectively. (**e**-**g**) The representative cross-sectional planes for software 1 (**e**), 2 (**f**), and 3 (**g**). Inner and outer lines (white and black for software 1; blue and green for software 2; orange and blue for software 3) represent the lumen and vessel boundaries, respectively. Software 1 = AutoSeg, AI Medic; Software 2 = Syngo.via, Siemens; Software 3 = IntelliSpace Portal, Philips

**Fig. 2:**
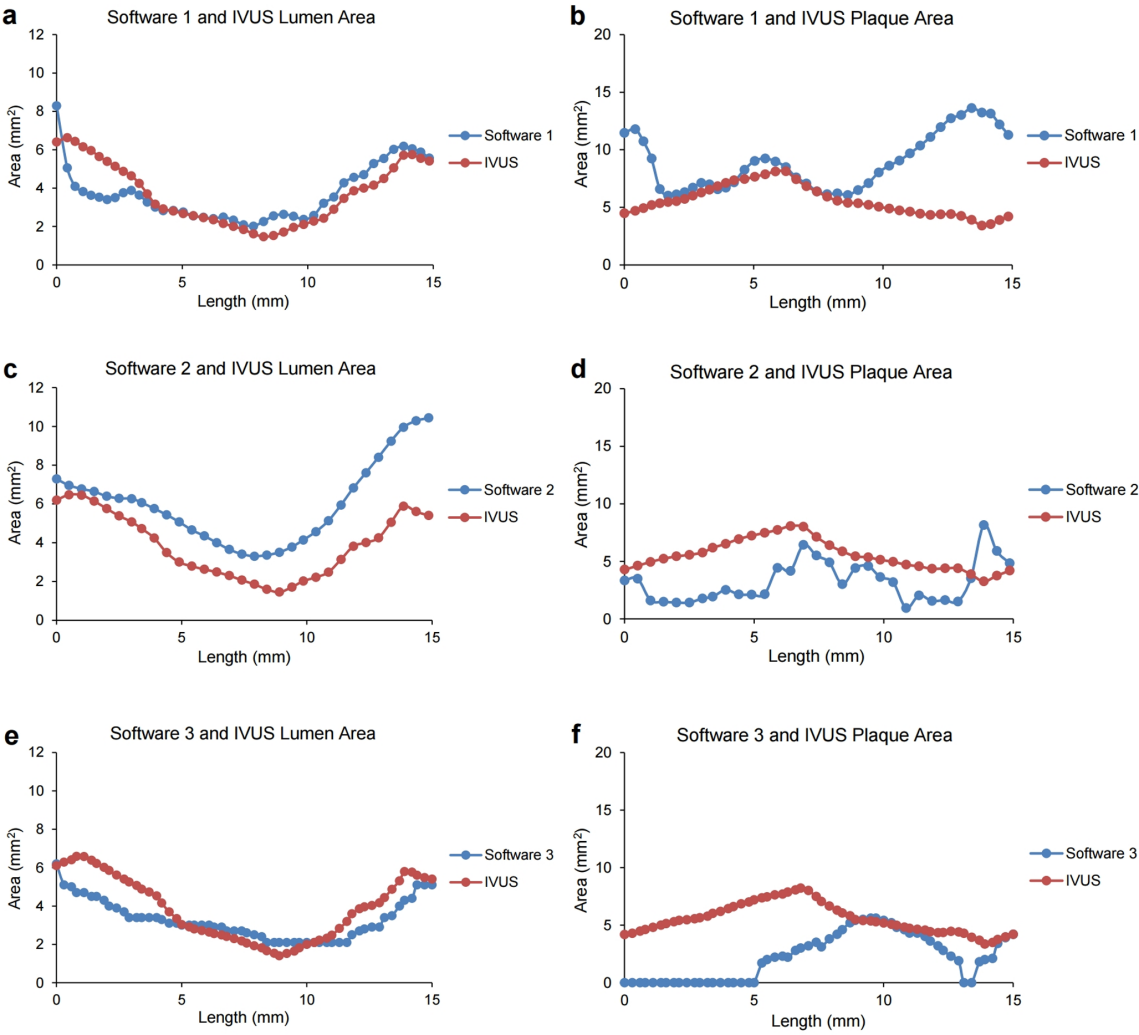
Representative lumen and plaque area graph plots for an individual patient measured by each software platform against the IVUS data. Graph plots of the lumen (**a**, **c**, and **e**) or plaque area (**b**, **d**, and **f**) measured by software 1 (**a** and **b**), 2 (**c** and **d**), or 3 (**e** and **f**) against the IVUS data. Software 1 = AutoSeg, AI Medic; Software 2 = Syngo.via, Siemens; Software 3 = IntelliSpace Portal, Philips

For each software platform available in Seoul National University Hospital (software 1 = AutoSeg, AI Medic; software 2 = Syngo.via, Siemens; software 3 = IntelliSpace Portal, Philips), fully automated coronary artery segmentation was performed without manual editing process. After coronary artery segmentation, CT-IVUS locations of the target vessels were matched using the provided information from IVUS, and the lumen or plaque area was measured for each cross-sectional plane by a specific interval using each software platform (Fig. 1 and Supplementary Table S2). The sample area graphs for an individual patient using each software platform are shown in Fig. 2. The longitudinal view of the segment analyzed in Fig. 2 is shown in Fig. 1. The interval of cross-sectional planes was different in IVUS data (0.001 mm) and software platforms (0.2–0.5 mm). Therefore, the IVUS data interval was adaptively modified to each software platform to compare the lumen or plaque area in each cross-sectional plane. The sum of the lumen or plaque areas multiplied by the specific interval of each software platform was used to calculate the lumen or plaque volume. The calculated volume was compared to the volume provided by the IVUS data for each target coronary segment.

### Deep learning-based location-adaptive threshold method for calculating lumen or plaque area

The lumen or plaque area was measured using a dedicated version of the software platform (AutoSeg-H ver. 1.05; AI Medic Inc., Seoul, South Korea). Note that, the software platform with DL-LATM was developed without using the CCTA images included in this study.

Centerlines and deep learning-derived calibration factors were automatically extracted from the software platform to segment coronary arteries. The software platform used artificial intelligence techniques and numerical algorithms in a hybrid manner to generate centerlines and deep learning-derived calibration factors as stated in Lee et al. [25].

Firstly, generating the centerlines requires a 3D model of the coronary artery. We utilized the V-net, a well-established fully convolutional neural network, for segmenting volumetric medical images [26–31]. The V-net was trained using manually segmented coronary artery CT images totaling 231,219 images.

Secondly, the calibration factors were derived from a 3D model of the aorta. For this, we employed Deeplab v3+, a widely-used neural network for semantic segmentation [32–35]. Training data for Deeplab v3 + consisted of manually segmented aorta CT images, totaling 103,076 images. Subsequently, calibration factors were extracted from the generated aorta using XGBoost, a commonly used system for addressing scale problems [25, 36–38].

Following the cross-sectional planes of the centerline, the lumen and vessel boundaries were separated by providing DL-LATM instead of a fixed Hounsfield Unit (HU) [9, 10]. The crucial characteristic for boundary detection lies in the attenuation difference relative to adjacent pixels. The algorithm, designed to locate lumen and outer vessel boundaries, searched for spots with the most significant change in attenuation in the direction from the inner point to the outer vessel. For lumen boundaries detection, we established upper and lower limits of attenuation. To exclude calcification, we chose the upper limit attenuation as 145% of the inner point, supported by a reference for detecting calcification using attenuation over 145% of the inner point [25]. For detecting the boundary of the coronary artery, the lower limit attenuation was set at 50% of the inner point. DL-LATM incorporates deep learning algorithms based on the LATM, which was previously published [21]. Since, LATM was developed based on the full width at half maximum, the lower limit attenuation was chosen as 50% of the inner point. The plaque region was decided by eliminating the lumen from outer vessel boundaries. However, if there was a big attenuation difference between the inner point and the calibration factor (inner point > calibration factor *1.45 or inner point < calibration factor * 0.5), the threshold was decided by the calibration factor. Similar to LATM, DL-LATM calculated lumen boundaries adaptively in each cross-sectional plane [21]. However, DL-LATM using the calibration factor could overcome the limitation of the existing LATM which overestimated lumen regions in the stenotic segment. Subsequently, the segmented cross-sectional planes were combined to make a 3D coronary artery model (Supplementary Figure S1).

### Subgroup lumen or plaque area analysis in the stenotic region

The given values of plaque burden from the IVUS data were used for subgroup analysis of the lumen or plaque area. The plaque burden is the ratio of the plaque area to the vessel area. The coronary lesions generally show high plaque burden with narrow lumen region surrounded by larger plaque components. For a subgroup analysis in the stenotic (plaque burden > 0.6) region, the lumen or plaque area was measured by three software platforms as compared to those derived by IVUS. The sample size of the subgroup is shown in Supplementary Table S3.

### Statistical analysis

For agreement analysis, the Bland-Altman plot was used to illustrate the bias against the difference in the measurements [39]. The limits of agreement were defined as the mean difference minus and plus 1.96 times the standard deviation (SD) of the differences for the lower and upper limits, respectively. In addition to Bland-Altman plot, the PCC and ICC were used to evaluate the reliability of each software against the IVUS data [40, 41]. The ICC was applied using two-way random effects, single measurement, and absolute agreement. To compare the correlation coefficient, Fisher’s r-to-Z transformation was used based on independent groups [42]. A p-value of 0.05 or less demonstrated a significant difference statistically. All analyses were performed using R version 4.2.0.

## Results

### Comparison of lumen or plaque volume for three different software platforms

To analyze the accuracy of the lumen or plaque segmentation using different software platforms, the calculated lumen or plaque volumes for each target segment were compared with the IVUS data. The statistical analysis of the PCC and ICC of the lumen or plaque volume for each software platform, using IVUS data as the gold standard, is shown in Table 1 and Supplementary Figure S2.

**Table 1:** Pearson correlation coefficient and intraclass correlation coefficient of the lumen or plaque volume for each software platform based on the IVUS data

		Software 1	Software 2	Software 3
Lumen volume	*r*†	0.92	0.91	0.92
	95% CI	0.83–0.97	0.78–0.96	0.83–0.97
	P value	< 0.001	< 0.001	< 0.001
	ICC	0.91	0.85	0.80
	95% CI	0.79–0.96	0.51–0.95	0.14–0.94
	P value	< 0.001	< 0.001	0.010
Plaque volume	*r*†	0.85	0.55	0.54
	95% CI	0.68–0.93	0.14–0.80	0.17–0.76
	P value	< 0.001	0.013	0.005
	ICC	0.71	0.55	0.41
	95% CI	0.35–0.87	0.15–0.79	-0.01-0.70
	P value	< 0.001	0.005	0.027

CI = confidence interval; ICC = intraclass correlation coefficient; *r*† = Pearson correlation coefficient; Software 1 = AutoSeg, AI Medic; Software 2 = Syngo.via, Siemens; Software 3 = IntelliSpace Portal, Philips

The PCCs for lumen volume were similar in all three software platforms, and software 1 showed the highest PCC for plaque volume (Table 1). The ICC was highest in software 1 for lumen and plaque volume (Table 1).

**Fig. 3:**
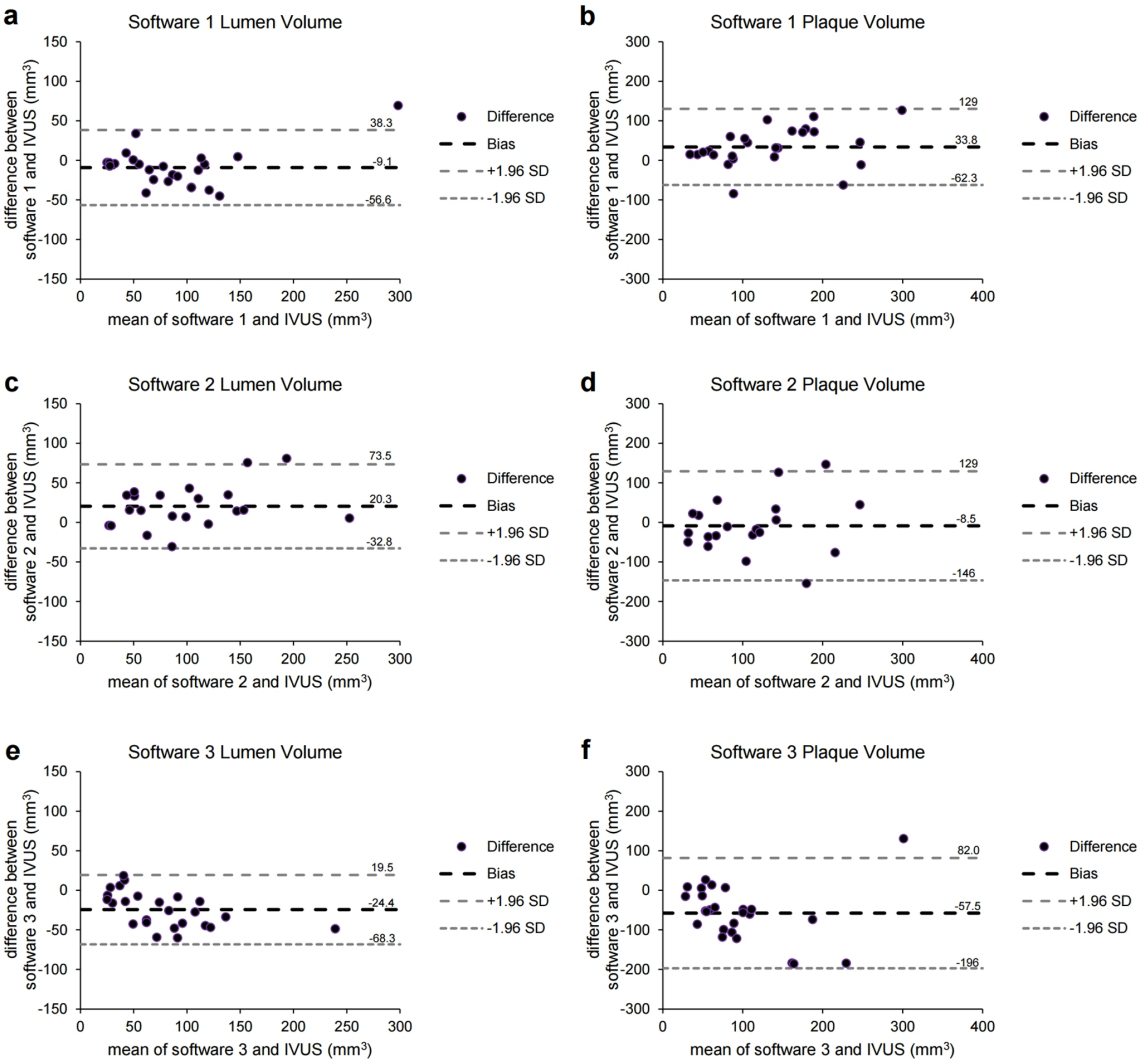
Bland-Altman plots of the lumen or plaque volume measured by differences between each software platform and the IVUS data. (**a**-**b**) Bland-Altman plots of the lumen (**a**) (mean difference = -9.1 mm^3^, SD = 24.2 mm^3^, 95% of CI of mean difference = -18.6 to 0.4 mm^3^) or plaque volume (**b**) (mean difference = 33.8 mm^3^, SD = 49.1 mm^3^, 95% of CI of mean difference = 14.6 to 53.0 mm^3^) measured by differences between software 1 and the IVUS data. (**c**-**d**) Bland-Altman plots of the lumen (**c**) (mean difference = 20.3 mm^3^, SD = 27.1 mm^3^, 95% of CI of mean difference = 8.7 to 31.9 mm^3^) or plaque volume (**d**) (mean difference = -8.5 mm^3^, SD = 70.3 mm^3^, 95% of CI of mean difference = -39.3 to 22.3 mm^3^) measured by differences between software 2 and the IVUS data. (**e**-**f**) Bland-Altman plots of the lumen (**e**) (mean difference = -24.4 mm^3^, SD = 22.4 mm^3^, 95% of CI of mean difference = -33.2 to -15.6 mm^3^) or plaque volume (**f**) (mean difference = -57.5 mm^3^, SD = 71.1 mm^3^, 95% of CI of mean difference = -84.7 to -30.1 mm^3^) measured by differences between software 3 and the IVUS data. CI = confidence interval; SD = standard deviation; Software 1 = AutoSeg, AI Medic; Software 2 = Syngo.via, Siemens; Software 3 = IntelliSpace Portal, Philips

The Bland-Altman plots for the lumen or plaque volume are shown in Fig. 3. Software 1 showed the bias closest to zero for lumen volume (mean difference = -9.1 mm^3^, SD = 24.2 mm^3^, 95% confidence interval [CI] = -18.6 to 0.4 mm^3^). Moreover, software 2 showed the bias closest to zero for plaque volume (mean difference = -8.5 mm^3^, SD = 70.3 mm^3^, 95% CI = -39.3 to 22.3 mm^3^), but SD was the lowest in software 1 (mean difference = 33.8 mm^3^, SD = 49.1 mm^3^, 95% CI = 14.6 to 53.0 mm^3^).

### Comparison of lumen or plaque area for three different software platforms

To analyze the accuracy of lumen or plaque segmentation using different software platforms with sufficient sample size, the measured lumen or plaque area was compared with IVUS data. The statistical analyses of the PCC and ICC of the lumen or plaque area for each software platform using IVUS data as the gold standard are shown in Table 2 and Supplementary Figure S3.

**Table 2:** Pearson correlation coefficient and intraclass correlation coefficient of the lumen or plaque area for each software platform based on the IVUS data

		Software 1	Software 2	Software 3
Lumen area	*r*†	0.79	0.75	0.76
	95% CI	0.77–0.80	0.73–0.78	0.73–0.78
	P value	< 0.001	< 0.001	< 0.001
	ICC	0.76	0.71	0.61
	95% CI	0.66–0.82	0.58–0.80	0.19–0.79
	P value	< 0.001	< 0.001	0.003
Plaque area	*r*†	0.50	0.22	0.31
	95% CI	0.47–0.53	0.16–0.27	0.26–0.36
	P value	< 0.001	< 0.001	< 0.001
	ICC	0.34	0.17	0.19
	95% CI	0.09–0.51	0.11–0.23	0.03–0.33
	P value	0.004	< 0.001	0.009

CI = confidence interval; ICC = intraclass correlation coefficient; *r*† = Pearson correlation coefficient; Software 1 = AutoSeg, AI Medic; Software 2 = Syngo.via, Siemens; Software 3 = IntelliSpace Portal, Philips

Software 1 showed the highest PCC and ICC values for the lumen and plaque areas (Table 2). The ICCs of software 1 for the lumen and plaque areas were significantly higher than those of other software platforms (*p* = 0.005 and *p* < 0.001, respectively) (Supplementary Table S4).

**Fig. 4:**
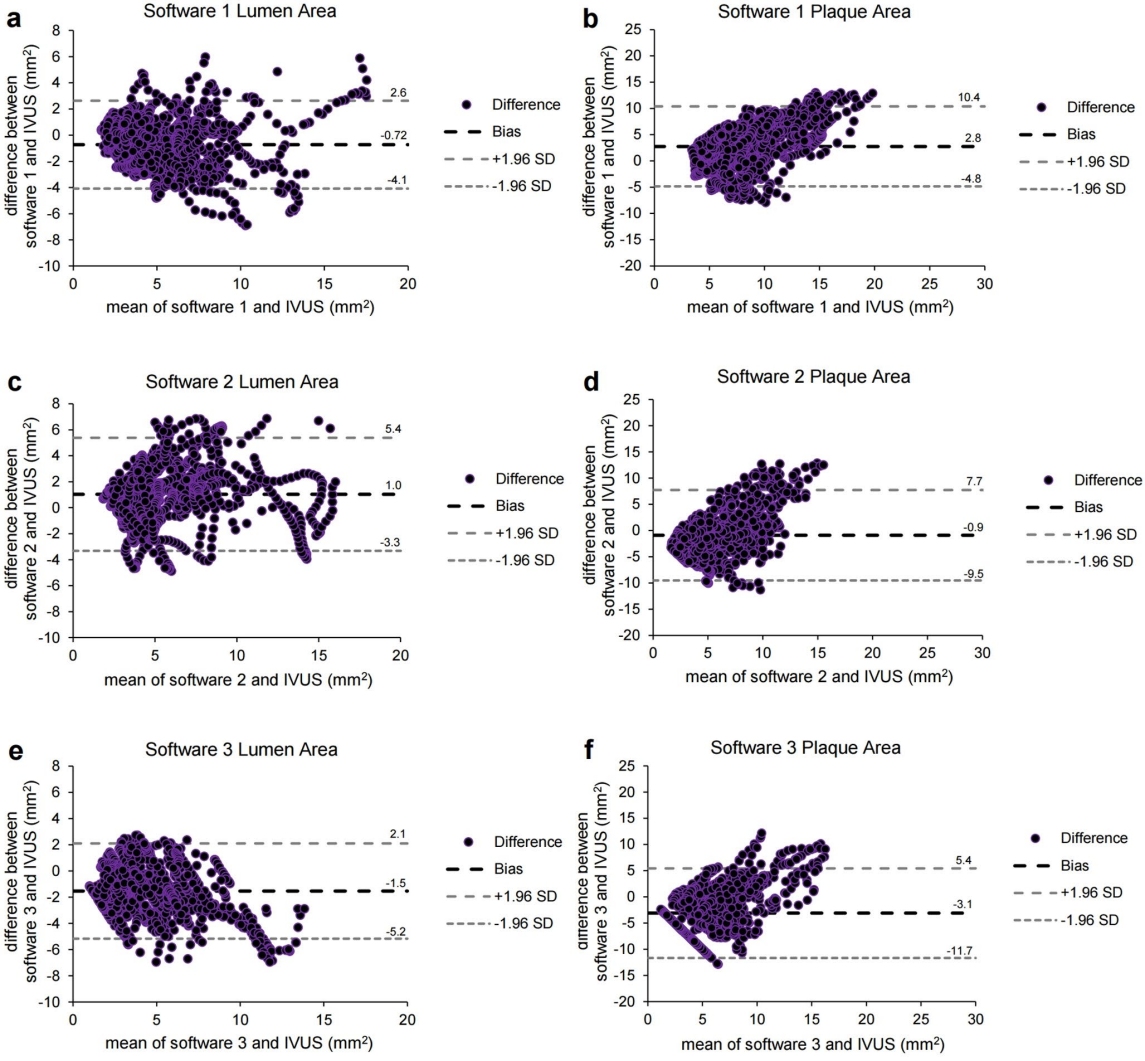
Bland-Altman plots of the lumen or plaque area measured by differences between each software platform and the IVUS data. (**a**-**b**) Bland-Altman plots of the lumen (**a**) (mean difference = -0.72 mm^2^, SD = 1.71 mm^2^, 95% of CI of mean difference = -0.80 to -0.64 mm^2^) or plaque area (**b**) (mean difference = 2.76 mm^2^, SD = 3.88 mm^2^, 95% of CI of mean difference = 2.58 to 2.94 mm^2^) measured by differences between software 1 and the IVUS data. (**c**-**d**) Bland-Altman plots of the lumen (**c**) (mean difference = 1.03 mm^2^, SD = 2.22 mm^2^, 95% of CI of mean difference = 0.90 to 1.16 mm^2^) or plaque area (**d**) (mean difference = -0.90 mm^2^, SD = 4.40 mm^2^, 95% of CI of mean difference = -1.17 to -0.63 mm^2^) measured by differences between software 2 and the IVUS data. (**e**-**f**) Bland-Altman plots of the lumen (**e**) (mean difference = -1.54 mm^2^, SD = 1.86 mm^2^, 95% of CI of mean difference = -1.64 to -1.44 mm^2^) or plaque area (**f**) (mean difference = -3.12 mm^2^, SD = 4.37 mm^2^, 95% of CI of mean difference = -3.36 to -2.88 mm^2^) measured by differences between software 3 and the IVUS data. CI = confidence interval; SD = standard deviation; Software 1 = AutoSeg, AI Medic; Software 2 = Syngo.via, Siemens; Software 3 = IntelliSpace Portal, Philips

Bland-Altman plots for the lumen or plaque area are shown in Fig. 4. Software 1 showed the bias closest to zero for the lumen area (mean difference = -0.72 mm^2^, SD = 1.71 mm^2^, 95% CI = -0.80 to -0.64 mm^2^). Moreover, software 2 showed the bias closest to zero for plaque area (mean difference = -0.90 mm^2^, SD = 4.40 mm^2^, 95% CI = -1.17 to -0.63 mm^2^), but SD was the lowest in software 1 (mean difference = 2.76 mm^2^, SD = 3.88 mm^2^, 95% CI = 2.58 to 2.94 mm^2^).

### Subgroup lumen or plaque area analysis in the stenotic region

Accurate segmentation of coronary lesions is important in the clinical view. Therefore, subgroup lumen or plaque area analysis in the stenotic (plaque burden > 0.6) region was performed using different software platforms in comparison with the IVUS data. Statistical analysis of the PCC and ICC of the lumen or plaque area in the stenotic region for each software platform using IVUS data as the gold standard is shown in Table 3 and Supplementary Figure S4.

**Table 3:** A subgroup analysis using Pearson correlation coefficient and intraclass correlation coefficient of the lumen or plaque area in the stenotic region for each software platform based on the IVUS data

		Software 1	Software 2	Software 3
Lumen area in stenotic region	*r*†	0.62	0.52	0.50
	95% CI	0.57–0.66	0.44–0.59	0.43–0.56
	P value	< 0.001	< 0.001	< 0.001
	ICC	0.61	0.31	0.46
	95% CI	0.56–0.65	0.00-0.53	0.33–0.56
	P value	< 0.001	0.025	< 0.001
Plaque area in stenotic region	*r*†	0.52	0.24	0.27
	95% CI	0.47–0.58	0.13–0.33	0.19–0.35
	P value	< 0.001	< 0.001	< 0.001
	ICC	0.36	0.15	0.16
	95% CI	0.25–0.44	0.02–0.27	0.05–0.27
	P value	< 0.001	0.012	0.002

CI = confidence interval; ICC = intraclass correlation coefficient; *r*† = Pearson correlation coefficient; Software 1 = AutoSeg, AI Medic; Software 2 = Syngo.via, Siemens; Software 3 = IntelliSpace Portal, Philips

Software 1 yielded the highest PCC and ICC values for the lumen and plaque areas (Table 3). The ICCs of software 1 for the lumen and plaque areas in the stenotic region were significantly higher than those of other software platforms (*p* < 0.001) (Supplementary Table S4).

**Fig. 5:**
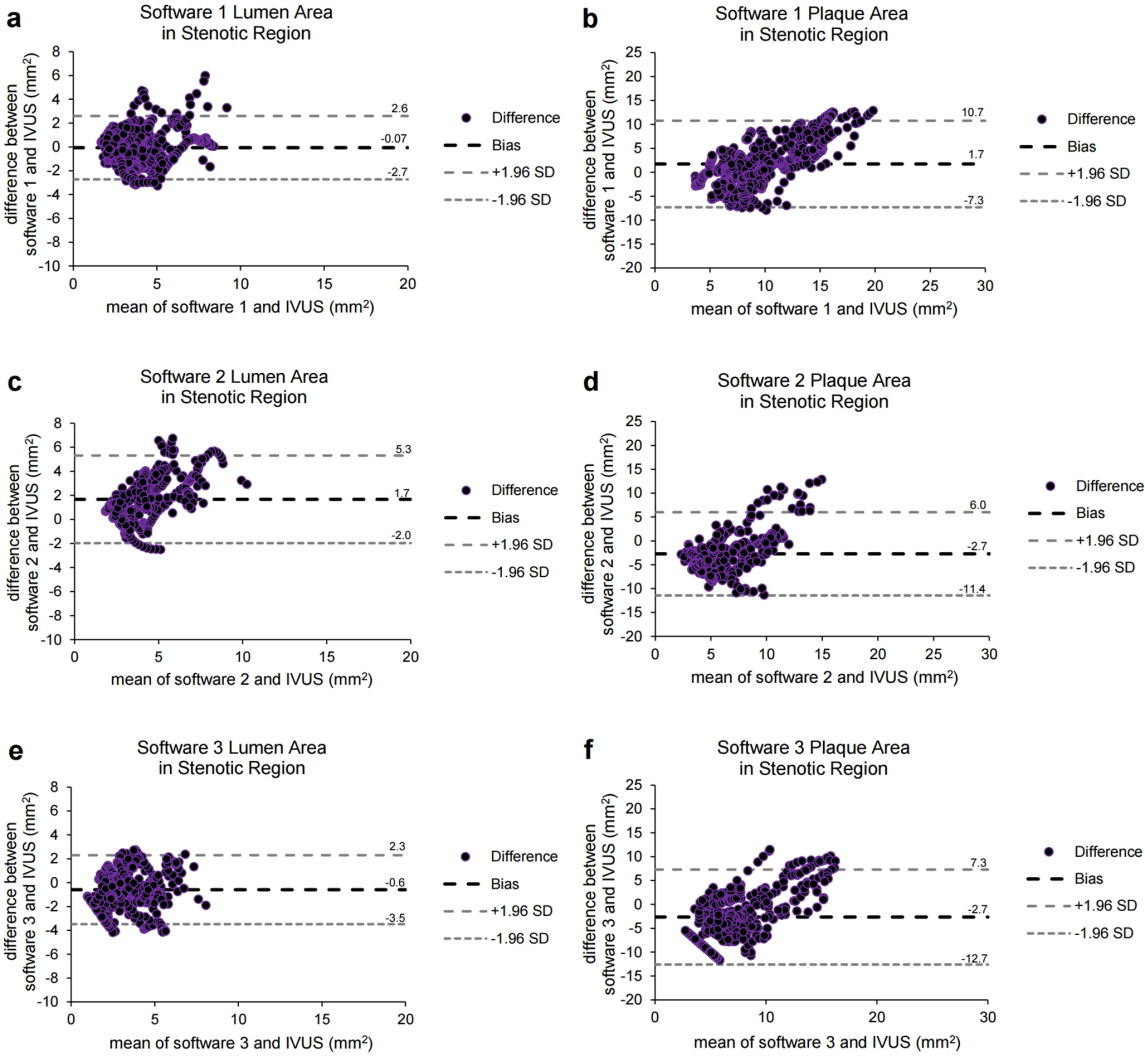
A subgroup analysis using Bland-Altman plots of the lumen or plaque area in the stenotic region measured by differences between each software platform and the IVUS data. (**a**-**b**) Bland-Altman plots of the lumen (**a**) (mean difference = -0.07 mm^2^, SD = 1.36 mm^2^, 95% of CI of mean difference = -0.16 to 0.02 mm^2^) or plaque area (**b**) (mean difference = 1.70 mm^2^, SD = 4.61 mm^2^, 95% of CI of mean difference = 1.37 to 2.03 mm^2^) in the stenotic region measured by differences between software 1 and the IVUS data. (**c**-**d**) Bland-Altman plots of the lumen (**c**) (mean difference = 1.66 mm^2^, SD = 1.86 mm^2^, 95% of CI of mean difference = 1.48 to 1.84 mm^2^) or plaque area (**d**) (mean difference = -2.72 mm^2^, SD = 4.45 mm^2^, 95% of CI of mean difference = -3.20 to -2.24 mm^2^) in the stenotic region measured by differences between software 2 and the IVUS data. (**e**-**f**) Bland-Altman plots of the lumen (**e**) (mean difference = -0.59 mm^2^, SD = 1.47 mm^2^, 95% of CI of mean difference = -0.72 to -0.46 mm^2^) or plaque area (**f**) (mean difference = -2.67 mm^2^, SD = 5.08 mm^2^, 95% of CI of mean difference = -3.11 to -2.23 mm^2^) in the stenotic region measured by differences between software 3 and the IVUS data. CI = confidence interval; SD = standard deviation; Software 1 = AutoSeg, AI Medic; Software 2 = Syngo.via, Siemens; Software 3 = IntelliSpace Portal, Philips

The Bland-Altman plots for the lumen or plaque area in the stenotic region are shown in Fig. 5. Software 1 showed the bias closest to zero for the lumen (mean difference = -0.07 mm^2^, SD = 1.36 mm^2^, 95% CI = -0.16 to 0.02 mm^2^) or plaque area (mean difference = 1.70 mm^2^, SD = 4.61 mm^2^, 95% CI = 1.37 to 2.03 mm^2^) in the stenotic region.

## Discussion

Automatic segmentation of coronary arteries using CCTA images can be accomplished using several commercially available software platforms, enabling analyses related to CAD [9, 10]. To analyze CAD more efficiently using software platforms, accurate segmentation of the coronary lumen or plaque in stenotic regions is essential. While several methods are available for coronary artery segmentation, they typically require manual segmentation processes to generate an accurate 3D model of coronary arteries, leading to intra- and inter- observer variability [11–13]. To overcome the laborious manual segmentation processes, several recent CCTA studies have utilized deep learning algorithms for coronary artery segmentation [14–16]. Deep learning approaches are emerging as promising analysis tools for CCTA imaging, and DL-LATM was developed using deep learning approaches combined with numerical algorithms. To determine the potential of DL-LATM in aiding CAD diagnosis, evaluating lumen and plaque segmentation performance was necessary. To this end, this study compared the segmentation performance of a software platform with DL-LATM (Software 1) against commercially available software platforms using the IVUS dataset as the gold standard.

Subgroup analysis of software platform segmenting coronary arteries led to the following conclusions: (i) Software 1 showed the highest PCC and ICC with the bias closest to zero for detecting lumen (PCC = 0.62, 95% CI = 0.57 to 0.66; ICC = 0.61, 95% CI = 0.56 to 0.65; mean difference = -0.07 mm^2^, 95% CI = -0.16 to 0.02 mm^2^) or plaque area (PCC = 0.52, 95% CI = 0.47 to 0.58; ICC = 0.36, 95% CI = 0.25 to 0.44; mean difference = 1.70 mm^2^, 95% CI = 1.37 to 2.03 mm^2^) in the stenotic region when compared with other software platforms based on IVUS data as the gold standard. (ii) Software 1 showed a significantly higher ICC for detecting the lumen or plaque area in the stenotic region than the other software platforms (*p* < 0.001). The proposed DL-LATM method could overcome the limitation of existing LATM which overestimated lumen boundaries in the stenotic region [21].

For all software platforms, lumen segmentation showed better performance than plaque segmentation because detecting the outer wall was generally more challenging than lumen segmentation [43]. In particular, software 2 showed an extremely large plaque area in some spots where coronary arteries were located near the chambers or veins, with a failure rate of 7% (Supplementary Figure S5 and Table S1). In contrast, software 3 showed an extremely small plaque area in some non-stenotic regions (Fig. 2f). Software 1 showed an overestimated tendency for plaque segmentation in non-stenotic region (Fig. 2b). To detect outer vessel boundaries, the attenuation of the spot should exhibit the most significant change compared to adjacent pixels and be less than 50% of the inner point HU. While this characteristic of the outer vessel boundaries is well demonstrated in the stenotic region with a high plaque burden, it may result in an overestimation of plaque area in the non-stenotic region with a low plaque burden. However, recognizing the importance of plaque segmentation in regions with a high plaque burden, software 1 showed the lowest bias of 1.7 mm^2^ for plaque area in the stenotic region compared with other software platforms (Fig. 5).

There are some limitations in this study. First, the sample size (*n* = 26) was relatively small. To overcome this limitation, we analyzed the lumen or plaque area in cross-sectional planes with a sufficient number to provide more detailed information (Supplementary Table S3). Second, there was an inconsistency between software platforms. The lumen or plaque area was compared with the IVUS data using the cross-sectional planes of the centerline for three software platforms. The interval of cross-sectional planes in IVUS data and three software platforms were 0.001 mm and 0.2–0.5 mm, respectively. Due to the interval differences among software platforms, the interval of the IVUS data was modified adaptively according to each software platform for area comparison. Nonetheless, the starting points and cross-sectional planes of the centerline in the reference data may not always precisely overlap with the CT images, potentially decreasing the overall correlation coefficient even though we precisely adjusted the interval of the reference data [22]. While this limitation might impact the overall correlation coefficient, there was no issue in comparing the segmentation performance of different software platforms.

In this study, our evaluation focused on segmentation performance in the stenotic region, allowing for a comparison of diagnostic significance among software platforms. Future applications may involve estimating the percentage of stenosis using coronary angiography as a reference. Additionally, comparing plaque types (such as calcification, lipid, etc.) across different software platforms can be explored in the future studies.

Moreover, the measurement of fractional flow reserve (FFR) is widely used to evaluate CAD [44, 45]. CAD patients with an FFR of 0.8 or less are recommended for stent implantation due to the potential for inducing ischemia [45, 46]. However, an invasive procedure is required to measure FFR [47]. CCTA-derived FFR (CT-FFR) is a non-invasive alternative technique used for CAD diagnosis [48–53]. Calculating CT-FFR requires the use of a 3D model of the coronary artery as primary input data, along with additional fluid dynamic techniques. The generated 3D coronary artery model from DL-LATM has the potential to serve as input data for CT-FFR calculation.

## Conclusions

The software platform with DL-LATM demonstrated reliable performance in detecting the lumen or plaque area in the stenotic region compared to commercially available software platforms. This study proposes a software platform using DL-LATM as an aid system for diagnosing CAD.

### Electronic supplementary material

Below is the link to the electronic supplementary material.

Supplementary Material 1

## Data Availability

The data set analyzed during the current study are not publicly available due to medical confidentiality but are available from the first author on reasonable request summarized form pending the approval of the IRB.

## Abbreviations

CAD: Coronary artery disease
CCTA: Coronary computed tomography angiography
CI: Confidence interval
CT-FFR: Coronary computed tomography angiography-derived FFR
FFR: Fractional flow reserve
HU: Hounsfield unit
ICC: Intraclass correlation coefficient
IVUS: Intravascular ultrasound
LATM: Location-adaptive threshold method
DL-LATM: Deep learning-based location-adaptive threshold method
PCC: Pearson correlation coefficient
SD: Standard deviation
