# Supplementary material for: A comparative analysis of deep learning-based location-adaptive threshold method software against other commercially available software
Source: Int J Cardiovasc Imaging. 2024 Apr 18;40(6):1269–81. doi: 10.1007/s10554-024-03099-7 (PMC11213768; doi:10.1007/s10554-024-03099-7)
Supplement: Supplementary file 1 — Supplementary Material 1 [file 10554_2024_3099_MOESM1_ESM.docx]

**Supplementary Material**

**A comparative analysis of deep learning-based location-adaptive threshold method software against other commercially available software**

**Contents**

**I. Supplementary Figures**

Supplementary Figure S1. A final coronary artery model based on deep learning- based location-adaptive threshold method.

Supplementary Figure S2. Pearson correlation coefficient plots of the lumen or plaque volume for each software platform based on the IVUS data.

Supplementary Figure S3. Pearson correlation coefficient plots of the lumen or plaque area for each software platform based on the IVUS data.

Supplementary Figure S4. Pearson correlation coefficient plots of the lumen or plaque area in the stenotic region for each software platform based on the IVUS data.

Supplementary Figure S5. A representatve coronary segment for the extremely large plaque area.

**II. Supplementary Tables**

Supplementary Table S1. The exclusion criateria and failure rates of coronary artery segmenation for different software platforms.

Supplementary Table S2. The characteristics of the target coronary segments

Supplementary Table S3. The number of cross-sectional planes of the coronary segments for different software platforms.

Supplementary Table S4. Correlation coefficient comparison of each software platform based on intraclass correlation coefficient.

**I. Supplementary Figures**

**Supplementary Figure S1. A final coronary artery model based on deep learning-based location-adaptive threshold method.**

(a) A final three-dimensional model of the coronary artery generated from the deep learning-based location-adaptive threshold method.

**Supplementary Figure S2. Pearson correlation coefficient plots of the lumen or plaque volume for each software platform based on the IVUS data.**

Pearson correlation coefficient plots of the lumen (a, c, and e) or plaque volume (b, d, and e) for software 1 (a and b), 2 (c and d), or 3 (e and f) based on the IVUS data. R^2^ = coefficient of determination; RMSE = root mean square error; Software 1 = AutoSeg, AI Medic; Software 2 = Syngo.via, Siemens; Software 3 = IntelliSpace Portal, Philips

**Supplementary Figure S3. Pearson correlation coefficient plots of the lumen or plaque area for each software platform based on the IVUS data.**

Pearson correlation coefficient plots of the lumen (a, c, and e) or plaque area (b, d, and f) for software 1 (a and b), 2 (c and d), or 3 (e and f) based on the IVUS data. R^2^ = coefficient of determination; RMSE = root mean square error; Software 1 = AutoSeg, AI Medic; Software 2 = Syngo.via, Siemens; Software 3 = IntelliSpace Portal, Philips

**Supplementary Figure S4. Pearson correlation coefficient plots of the lumen or plaque area in the stenotic region for each software platform based on the IVUS data.**

Pearson correlation coefficient plots of the lumen (a, c, and e) or plaque area (b, d, and f) in the stenotic region for software 1 (a and b), 2 (c and d), or 3 (e and f) based on the IVUS data. R^2^ = coefficient of determination; RMSE = root mean square error; Software 1 = AutoSeg, AI Medic; Software 2 = Syngo.via, Siemens; Software 3 = IntelliSpace Portal, Philips

**Supplementary Figure S5.** **A representatve coronary segment for the extremely large plaque area.**

A curved planar reformation image (a) and plaque area graph of software 2 and IVUS data (b) using a representative coronary segment. Software 2 = Syngo.via, Siemens

**II. Supplementary Tables
Supplementary Table S1. The exclusion criateria and failure rates of coronary artery segmenation for different software platforms.**

|  | Exclusion criteria (Difference between the results measured by a software platform and IVUS) | Failure rates (%) | | |
| --- | --- | --- | --- | --- |
|  |  | Software 1 | Software 2 | Software 3 |
| File open | DICOM import failure | 0.0 | 21.5 | 0.0 |
| Lumen volume | 100 mm^3^ | 3.8 | 0.0 | 3.8 |
| Plaque volume | 200 mm^3^ | 3.8 | 4.8 | 0.0 |
| Lumen area | 7 mm^2^ | 1.6 | 0.7 | 1.2 |
| Plaque area | 13 mm^2^ | 1.6 | 7.0 | 0.8 |

DICOM = Digital Imaging and Communications in Medicine; Software 1 = AutoSeg, AI Medic; Software 2 = Syngo.via, Siemens; Software 3 = IntelliSpace Portal, Philips

**Supplementary Table S2. The characteristics of the target coronary segments.**

|  |  | Characteristics | | | | | |
| --- | --- | --- | --- | --- | --- | --- | --- |
|  | Location of the lesion | Measurement direction | Length (mm) | Plaque volume measured in IVUS (mm^3^) | Coronary artery calcium score on CCTA | Stenosis degree (%) | Plaque burden |
| Segment #1 | LAD, just distal D1 | Downstream | 7 | 36.1 | N/A | 25-49 | 0.44 -0.57 |
| Segment #2 | LAD, just distal D2 | Downstream | 12 | 45.4 | 0 | 1-24 | 0.35-0.68 |
| Segment #3 | RCA just proximal RV2 | Upstream | 15 | 138.8 | 1 | 25-49 | 0.42-0.79 |
| Segment #4 | LAD, just distal D1 | Downstream | 15 | 81.5 | N/A | 50-69 | 0.23-0.85 |
| Segment #5 | LAD, just proximal D2 | Upstream | 15 | 75.1 | 37.2 | 70-99 | 0.30-0.78 |
| Segment #6 | RCA, just distal RV | Downstream | 25 | 124.6 | 0 | 25-49 | 0.34-0.75 |
| Segment #7 | LCX, just distal Os | Downstream | 40 | 253.7 | 0 | 1-24 | 0.37-0.88 |
| Segment #8 | LAD, just distal Os | Downstream | 15 | 83.5 | 0 | 70-99 | 0.37-0.79 |
| Segment #9 | RCA, just proximal RV | Upstream | 18 | 139.4 | 52 | 50-69 | 0.41-0.77 |
| Segment #10 | LAD, just distal D1 | Downstream | 20 | 135.3 | 317.9 | 25-49 | 0.46-0.77 |
| Segment #11 | LCX, just distal Os | Downstream | 7 | 26.5 | 5.11 | 50-69 | 0.38-0.68 |
| Segment #12 | LAD, just distal D1 | Downstream | 22 | 235.8 | 821.2 | 25-49 | 0.58-0.80 |
| Segment #13 | RCA, just proximal RV | Upstream | 20 | 135.6 | 0 | 25-49 | 0.30-0.63 |
| Segment #14 | RCA, just distal RV2 | Downstream | 11 | 79.7 | N/A | 50-69 | 0.40-0.86 |
| Segment #15 | LAD, just distal D2 | Downstream | 12 | 54.7 | N/A | 50-69 | 0.25-0.53 |
| Segment #16 | LAD, just distal D1 | Downstream | 20 | 153.3 | 39.9 | 25-49 | 0.56-0.66 |
| Segment #17 | LAD, just proximal D1 | Upstream | 7.8 | 86.8 | 0 | 50-69 | 0.68-0.80 |
| Segment #18 | LAD, just distal D1 | Downstream | 20 | 128.4 | 62.3 | 25-49 | 0.50-0.60 |
| Segment #19 | LCX, just distal early OM | Downstream | 10 | 130.5 | 66.7 | 70-99 | 0.51-0.83 |
| Segment #20 | LAD, just distal D1 | Downstream | 10 | 40.1 | 84.5 | 50-69 | 0.46-0.52 |
| Segment #21 | RCA, just distal RV2 | Downstream | 30 | 321.6 | 186.5 | 50-69 | 0.34-0.76 |
| Segment #22 | LCX, just distal Os | Downstream | 15 | 125.6 | 0 | 1-24 | 0.42-0.67 |
| Segment #23 | LAD, just distal Os | Downstream | 27 | 224.1 | 88.9 | 50-69 | 0.27-0.79 |
| Segment #24 | LCX, just distal Os | Downstream | 10 | 86.1 | 16 | 25-49 | 0.27-0.58 |
| Segment #25 | LM, just proximal LCX Os | Upstream | 8 | 56.5 | 0 | 50-69 | 0.54-0.79 |
| Segment #26 | RCA, just proximal RV2 | Upstream | 20 | 76.0 | 0 | 1-24 | 0.40-0.54 |

CCTA = Coronary computed tomography angiography; D = diagonal branch; LAD = left anterior descending artery; LCX = left circumflex artery; LM = left main coronary artery; N/A = not available; OM = obtuse marginal branch; Os = ostium; RV1 = sinoatrial artery; RV2 = conus artery

**Supplementary Table S3. The number of cross-sectional planes of the coronary segments for different software platforms.**

|  | Number of cross-sectional planes | | |
| --- | --- | --- | --- |
|  | Software 1 | Software 2 | Software 3 |
| Lumen area | 1795 | 1100 | 1313 |
| Plaque area | 1795 | 1029 | 1319 |
| Lumen area in stenotic region | 725 | 390 | 516 |
| Plaque area in stenotic region | 730 | 332 | 514 |

Software 1 = AutoSeg, AI Medic; Software 2 = Syngo.via, Siemens; Software 3 = IntelliSpace Portal, Philips

**Supplementary Table S4. Correlation coefficient comparison of each software platform based on intraclass correlation coefficient.**

|  | ICC | | | ICC comparison (p-value) | | |
| --- | --- | --- | --- | --- | --- | --- |
|  | S1 ICC | S2 ICC | S3 ICC | S1 vs. S2 | S1 vs. S3 | S2 vs. S3 |
| Lumen volume | 0.91  (0.79-0.96) | 0.85  (0.51-0.95) | 0.80  (0.14-0.94) | 0.393 | 0.155 | 0.620 |
| Plaque volume | 0.71  (0.35-0.87) | 0.55  (0.15-0.79) | 0.41  (-0.01-0.70) | 0.405 | 0.130 | 0.568 |
| Lumen area | 0.76  (0.66-0.82) | 0.71  (0.58-0.80) | 0.61  (0.19-0.79) | 0.005 | <0.001 | <0.001 |
| Plaque area | 0.34  (0.09-0.51) | 0.17  (0.11-0.23) | 0.19  (0.03-0.33) | <0.001 | <0.001 | 0.620 |
| Lumen area in stenotic region | 0.61  (0.56-0.65) | 0.31  (0.00-0.53) | 0.46  (0.33-0.56) | <0.001 | <0.001 | 0.009 |
| Plaque area in stenotic region | 0.36  (0.25-0.44) | 0.15  (0.02-0.27) | 0.16  (0.05-0.27) | <0.001 | <0.001 | 0.885 |

Data represented in parentheses are 95% confidence interval; ICC = intraclass correlation coefficient; S1 = AutoSeg, AI Medic; S2 = Syngo.via, Siemens; S3 = IntelliSpace Portal, Philips
